# Intra- and interspecies competition of blackgrass and wheat in the context of herbicidal resistance and environmental conditions in Poland

**DOI:** 10.1038/s41598-022-12777-2

**Published:** 2022-05-24

**Authors:** Anna Wenda-Piesik, Agnieszka Synowiec, Katarzyna Marcinkowska, Barbara Wrzesińska, Cezary Podsiadło, Krzysztof Domaradzki, Piotr Kuc, Ewa Kwiecińska-Poppe

**Affiliations:** 1 Department of Agronomics, Faculty of Agriculture and Biotechnology, Bydgoszcz University of Science and Technology, Al. Kaliskiego 7, 85-796 Bydgoszcz, Poland; 2 Department of Agroecology and Crop Production, Faculty of Agriculture and Economics, University of Agriculture in Krakow, Al. Mickiewicza 21, 31-120 Kraków, Poland; 3 Institute of Plant Protection—National Research Institute, ul. Władysława Węgorka 20, 60-318 Poznań, Poland; 4 Department of Agroengineering, The West Pomeranian University of Technology in Szczecin, ul. Papieża Pawła VI 3, 71-459 Szczecin, Poland; 5 Department of Weed Science and Soil Tillage Systems, Institute of Soil Sciences and Plant Cultivation—State Research Institute, Orzechowa 61, 50-540 Wrocław, Poland; 6 Institute of Agroecology and Plant Production, Wrocław University of Environmental and Life Sciences, pl. Grunwaldzki 24A, 50-363 Wrocław, Poland; 7 Department of Herbology and Plant Cultivation Techniques, University of Life Sciences in Lublin, Akademicka 13, 20-950 Lublin, Poland

**Keywords:** Plant sciences, Environmental sciences

## Abstract

Blackgrass (*Alopecurus myosuroides* Huds.), one of the most aggressive grass weeds in Europe, is also a strong competitor of crops. This study aimed to assess the impact of environmental conditions on the competition between (1) ACC-ase and ALS herbicide-resistant (BR) and herbicide-susceptible (BS) blackgrass biotypes, (2) BR and winter wheat *cv*. Arkadia (W), and (3) BS and W. In the replacement series model, the experiment was conducted at seven sites across Poland during two seasons (2018/19 and 2019/20). In the BR-BS experiment, the BS biotype was in majority more competitive toward the BR biotype. However, in the regime of optimal hydrothermal conditions and at a higher sand content in the soil we observed a higher competitiveness of BR towards BS. The combined interactions between W and BR or BS were also affected by environmental conditions, i.e., soil texture and hydrothermal coefficient, as explained by PCA and k-*means* cluster analysis. At most sites, W was more competitive toward both BS and BR, which could result from earlier emergence of W in relation to B in majority of sites. Except for two cases, located on heavy, clay soils, during humid seasons, where B was more competitive toward W. We summarize that blackgrass competitiveness towards other biotypes and wheat depends to some extent on environmental conditions; however, the phenomenon should be explored in more detail.

## Introduction

Blackgrass, *Alopecurus myosuroides* Huds., is a winter annual, monocotyledonous plant that produces many seeds and is one of the most difficult weeds to control^1–3^. Fields with consistently dense *A. myosuroides* patches are characterized by high clay, nutrient, and organic matter contents with a slightly lower pH^4–6^. Blackgrass emerges in early autumn, often before the emergence of winter crops, i.e., cereals and oilseed rape^7,8^. A phenotypic variation of blackgrass and its dormancy affect its emergence patterns in a single year, bringing this weed's evolutionary advantage over crops^9,10^. During the last 50 years, blackgrass has become more prevalent and problematic to control weeds, mostly due to widespread herbicide resistance^11^. The first case of herbicide-resistant blackgrass was reported in 1982 in Great Britain^12^. Since then, the weed has evolved field resistance to a few different herbicide modes of action^13^, especially to post-emergence acetyl-CoA carboxylase inhibitors (aryloxyphenoxypropionate (AOPP) and cyclohexanedione (CHD) and acetolactate synthase (ALS) inhibitors^14^. The mechanism behind blackgrass resistance is reported to be enhanced metabolism in the case of resistance to AOPP/CHD herbicides^15^ and target-site resistance (TSR) in the case of ACCase-inhibiting and ALS-inhibiting herbicides^16,17^. Non-target-site-based resistance (NTSR) mechanisms in blackgrass can confer unpredictable resistance to herbicides with different chemistries or modes of action^18–21^, but no glyphosate resistance has been reported to date^22^. The UK's latest report showed that the herbicide resistance-prone, outcrossing *A. myosuroides* exhibits low genetic differentiation among field-collected populations, with some geographical (latitudinal and longitudinal) clines in genetic diversity and isolation by distance^23^. In Poland, herbicide-resistant blackgrass populations are mostly found in winter wheat, especially in the northeastern parts of the country^24–26^.

Blackgrass is a very competitive weed for winter wheat that can cause grain yield losses of up to 41%^8^. At a density of 25 plants m^−2^, a 10% decrease in wheat yield is observed^27^. Studies on wheat-blackgrass competitiveness could support integrated weed management techniques, in addition to mechanical, biological, and cultural control means^3,28^. Wheat planting design, e.g., plant density, row spacing, and orientation, increases wheat competitiveness against weeds^29–31^, which is one of the methods of weed management. In the case of the herbicide-resistance trait in agrocenosis, what counts is the competitive pressure of resistant weeds on crops and the interspecies competitiveness of resistant biotypes towards susceptible ones^32,33^. Identifying this interaction type could be important, especially when herbicide resistance has developed in the field. Nevertheless, due to the introduction of sustainable weed management methods, herbicides have substantially reduced. In that situation, weeds of different susceptibilities to herbicides could compete with each other^33,34^. The available literature on that topic shows that the competition between resistant vs susceptible biotype depends on many factors, i.e., type of resistance, habitat stresses, and weed densities^32–35^. On the other hand, the cited studies were performed either in the controlled conditions or in one field site only. For these reasons, testing the resistant vs susceptible biotype in different climatic conditions is a novel aspect of our research. The methods developed to study competition among plants allow the estimation of the losses caused by weeds and the competitiveness among species^36^. The models for blackgrass and wheat competition may differ significantly in predicting the yield damage caused by blackgrass, and there is no optimum design for competition experiments since the aims, objectives, and practicalities vary from study to study and species to species. However, they could potentially be used in future weed management systems^37,38^. Our study focused on a replacement-series competition model^39^ to study the competitive abilities of herbicide-resistant blackgrass toward the susceptible biotype and toward winter wheat cv. Arkadia. A standard replacement series comprises a set of pure and mixed populations in which the combined density of the components is held constant^40^. The method was developed in 1960 by de Wit^41^. Correctly used, the approach can lead to some valid interpretations. However, this methodology has some limitations, i.e., a replacement series is unsuitable for the quantitative evaluation of interference or niche differentiation or in predicting the long-term outcome of an association between species^40^. Based on the replacement series design, a recent study showed that the multiple herbicide-resistant bentgrass (*Apera spica-venti* (L.) P. Beauv) was more competitive toward winter wheat than the susceptible bentgrass^42^.

This study aimed to assess the impact of habitat conditions on the competition between (1) herbicide-resistant and herbicide-susceptible blackgrass biotypes, (2) resistant blackgrass and winter wheat, and (3) susceptible blackgrass and winter wheat. We therefore hypothesized that both habitat conditions, species/biotype frequencies, and herbicide resistance of blackgrass affect the competition of plants.

## Results

### Confirmation of blackgrass biotype resistance to ALS and ACCase inhibitors

**Figure 1:**
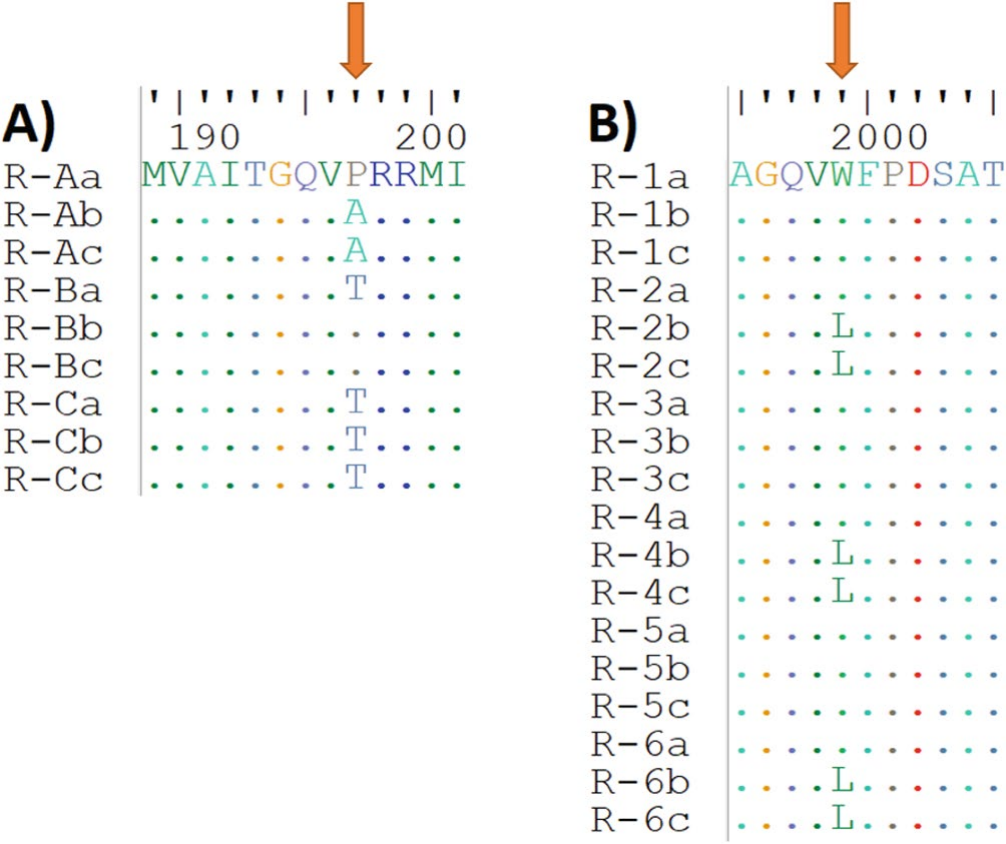
The alignment of blackgrass ALS (**A**) and ACCase (**B**) amino acid sequence fragments derived from the plants belonging to the R biotype. The red arrow indicates the mutations known to confer herbicide resistance to ALS or ACCase inhibitors. (**A**) The uppercase letters (A, B, or C) in sequence names represent DNA templates used in PCR—consisting of pooled equal amounts of DNA isolated from two plants. Amino acid numbering refers to the blackgrass ALS sequence (**B**). The numbers in the sequence names represent the plant number. The amino acid numbering refers to blackgrass ACCase sequence, a–c—clone name.

The amplification of the blackgrass ALS sequence resulted in the generation of a 1919 bp nucleotide sequence. Mutations were identified in the analyzed nucleotide sequence fragment, where 22 changes were synonymous and seven were nonsynonymous. The analysis of the amino acid sequence to find mutations known to confer resistance to ALS inhibitors revealed the presence of an amino acid substitution at position P197. Two mutations were found: P197A and P197T (Fig. 1A).

The amplification of the blackgrass ACCase sequence fragment resulted in the generation of 2879 bp nucleotide sequences comprising nucleotides from 9223 to 120,101 in the ACCase sequence. The analyzed fragment contained a DNA sequence encoding the carboxyl-transferase (CT) domain (nucleotides from 1117 to 2765 of the analyzed PCR product), which is known to be the site of action for the active substance of the herbicides belonging to group 1. Four mutations were identified in the analyzed nucleotide sequence of the CT domain, where three changes were synonymous and one was nonsynonymous. The nonsynonymous mutation resulted in the substitution of Trp to Leu at position 1999 in the amino acid sequence (Fig. 1B), which is a mutation known for conferring resistance to ACCase inhibitors.

### Competition between resistant and susceptible biotypes of blackgrass

ANOVA effects for the relative plant biomass (BCRb) and the relative grain number (BCRgn) of S and R blackgrass biotypes are presented in Table 1. The single effect for site and the interaction effect for year × site were significant for both BCRb and BCRgn, while BCRgn was also significantly affected by the single effect of year.

**Table 1:** Mean squares from the three-way analysis of variance for the competitive ratio of susceptible (S) and herbicide-resistant blackgrass (R) calculated for the relative plant biomass (BCRb) and relative grain number (BCRgn).

Source of variation	*df*	BCRb	BCRgn
Year	1	0.01	0.91**
Site	6	0.33***	0.81***
Year × site	6	0.27***	0.72***
Residual	60	0.06	0.13

*df* degrees of freedom.

*P < 0.05; **P < 0.01; ***P < 0.001.

The values of BCRb (BCRgn) < 1 indicate that the R biotype is more competitive toward the S biotype in the case of biomass (grain) production. In contrast, if BCRb (BCRgn) > 1, then the S biotype is more competitive toward R.

**Figure 2:**
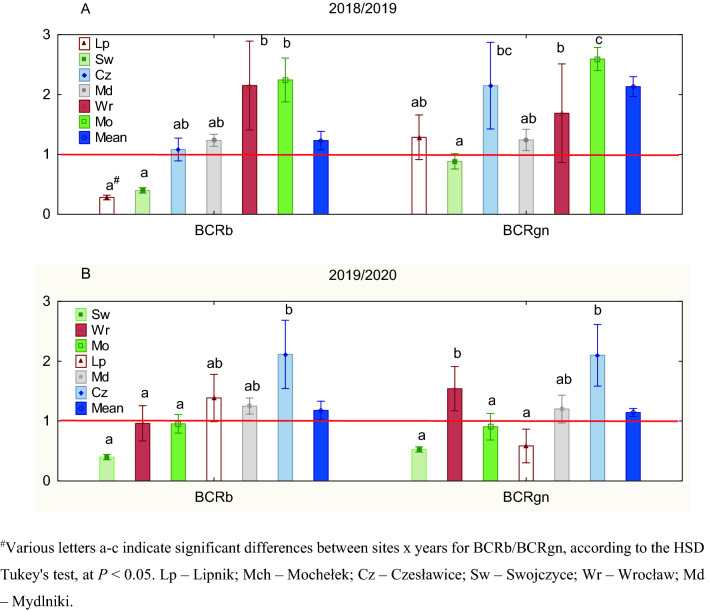
Competitive ratio for plant biomass (BCRb) and grain (BCRgn) between susceptible and herbicide-resistant blackgrass biotypes in the years of study for six sites in Poland. Mean (*n* = 6) ± standard error. Competitive ratio values < 1 indicate that the herbicide-resistant biotype of blackgrass is more competitive than the susceptible biotype.

**Figure 3:**
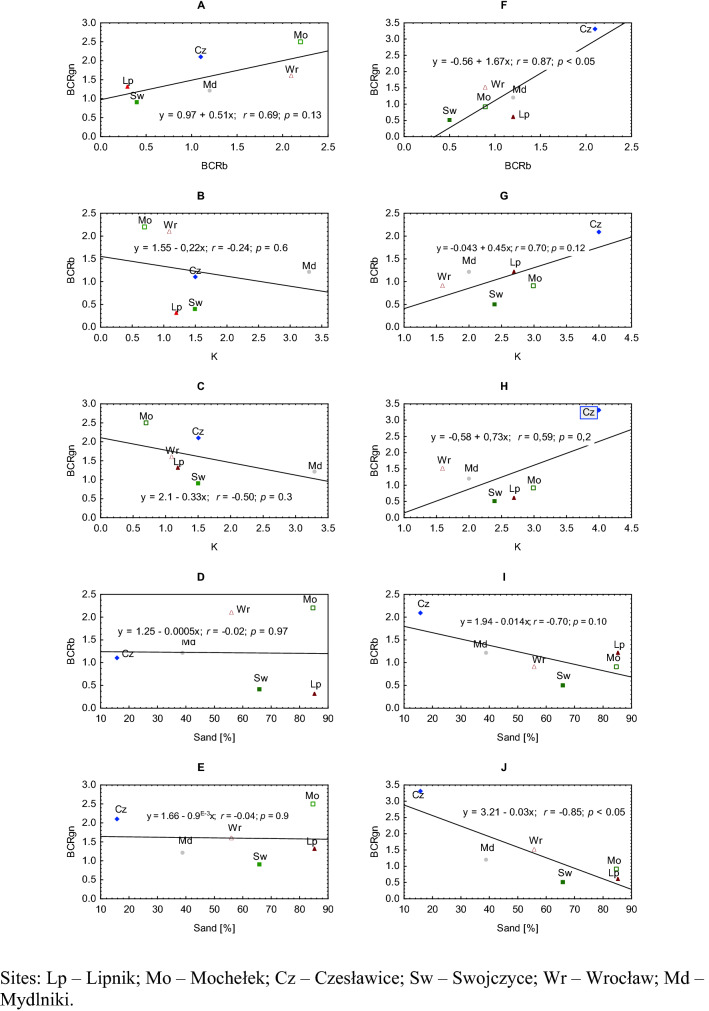
Correlation coefficients (r-Pearson) and linear regression of competitive ratio for biomass (BCRb) and grain numbers (BCRgn) between susceptible and herbicide-resistant blackgrass and between hydrothermal coefficient (K), sand content, and BCRb and BCRgn in two years of study (**A**–**E**)—2018/2019, (**F**–**J**)—2019/2020.

A detailed site × year comparison showed that BR and BS biotypes competed differently. During 2018/19, the BR biotype was more competitive in BCRb at two sites, i.e., Lipnik and Swojczyce, and in BCRgn at one site, i.e., Swojczyce (Fig. 2A). The BS biotype was also more competitive toward BR in BCRb in two sites, i.e., Wrocław and Mochełek, and in BCRgn in all the sites, except for Swojczyce. There was a positive correlation (*r* = 0.69) between BCRb and BCRgn in 2018/19, but the correlation was nonsignificant (Fig. 3A). In terms of the correlation of each competitive ratio in 2018/2019 with the hydrothermal coefficient K, a negative correlation was found, especially for BCRgn (*r* = − 0.50) (Fig. 3B,C), indicating that the drier the weather was, the more competitive the BS biotype; however, the trends were not significant. There was no correlation found for the competitive ratios and the soil conditions, expressed here as a percentage content of sand in the soil (Fig. 3D,E).

In the 2019/20 season, the average BCRgn value was significantly, by 38%, lower than that in 2018/19, whereas the value of BCRb was similar (Fig. 2B). During that season, the BR biotype was more competitive toward BS again in Swojczyce but also in Mochełek and in terms of BCRb in Wrocław and BCRgn in Lipnik. In this season, both the competitive ratios BCRb and BCRgn were positively (*r* = 0.87) and significantly correlated (Fig. 3F). The hydrothermal conditions K indicated mostly humid conditions in Poland in the 2019/20 season (Table 9, Materials and methods section). The *r*-Pearson correlations between the K coefficient and the BCRb (BCRgn) indexes had the opposite direction (*r* = 0.7 and 0.59, respectively) as in the dry season of 2018/2019. The increasing K provoked an increase in the competitiveness of the S biotype, both in biomass and grain production (Fig. 3G,H). On the other hand, a negative correlation occurred in the 2019/2020 season between the sand content [%] in the soil and BCRb (BCRgn), *r* = − 0.70 and *r* = − 0.85 (significant), respectively (Fig. 3 I,J). The higher the sand content in the soil was, the higher the competitiveness of the BR biotype towards BS.

### Competition between winter wheat and resistant/susceptible biotypes of blackgrass

The emergence of winter wheat (W) was noted 8–16 and 6–19 days after sowing in 2018 and 2019, respectively (Table 2). The fastest emergence of W was noted in 2018 in Mydlniki and Lipnik and the longest in Mochełek and Czesławice. Even though the dates of sowing for both W and B were the same, B emerged, 0–15 days later than W at most sites. In 2018, the emergence of B on the same day as W occurred in Lipnik and Mochełek, while in Winna Góra and Mydlniki, B emerged 6 days after W. In autumn 2019, the emergence of W and B was at the same date in Mochełek and Mydlniki (Table 2).

**Table 2:** Dates of winter wheat and blackgrass emergence, number of days from wheat sowing until emergence, and the emergence of blackgrass to wheat.

Site	Autumn 2018				Autumn 2019			
	W^1^	Days to W emergence	B^2^	The emergence of B to W (average, days)	W	Days to W emergence	B	The emergence of B to W (average, days)
Lipnik	25.10	8	25.10	0	25.10	9	29.10	+ 4
Mochełek	02.11	16	02.11	0	02.11	16	02.11	0
Winna Góra	30.10	12	05.11	+ 6	16.11	16	31.10	+ 15
Czesławice	23.10	15	25.10	+ 2	24.10	15	26.10	+ 2
Swojczyce	23.10	10	26.10	+ 3	05.11	19	26.11	+ 11
Wrocław	05.11	11	08.11	+ 3	14.10	6	18.10	+ 4
Mydlniki	12.10	8	18.10	+ 6	24.10	18	24.10	0

^1^*W* winter wheat.

^2^*B* blackgrass.

The ANOVA for WBCRgn and WBCRb displays significant effects for competitive ratios related to the site and the interaction year × site (Table 3). Regarding WBCRgn, a significant effect was also detected in the interaction site × biotype. For WBCRb, the interaction of year × biotype × site was also significant.

**Table 3:** Mean squares from the three-way analysis of variance for the competitive ratio of winter wheat (W) and herbicide-resistant or herbicide-susceptible blackgrass (B) calculated for the relative grain number (WBCRgn) and plant biomass (WBCRb).

Source of variation	*df*	WBCRgn	WBCRb
Year	1	2.87	0.67
Site	6	10.9***	11.7***
Blackgrass biotype	1	1.20	0.22
Year × site	6	2.62**	1.54**
Year × biotype	1	0.02	2.94**
Site × biotype	6	2.45***	0.27
Year × biotype × site	6	1.18	3.19***
Residual	140	0.81	0.36

*df* degrees of freedom.

*P < 0.05; **P < 0.01; ***P < 0.001.

**Figure 4:**
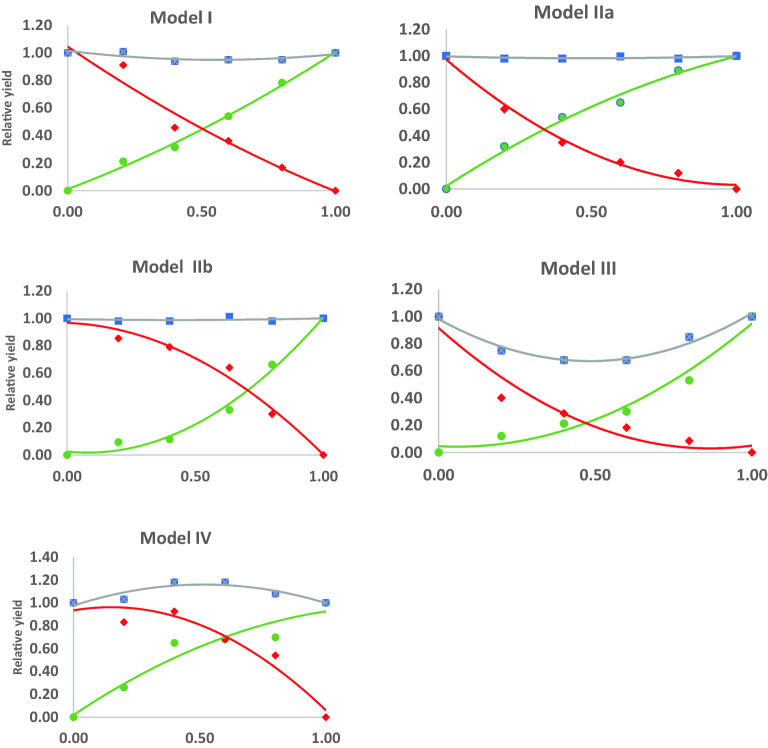
The models of competition between winter wheat (W) and blackgrass (B) for the replacement series experiments. The vertical axis represents relative values of a grain number or biomass, whereas the horizontal axis represents the proportion (0 to 1) of species in a mixture. Legend: green line—B; red line—W, grey line—B + W.

The individual model of competition between blackgrass and winter wheat for each site, both R and S biotypes, and during two seasons was confirmed by the χ^2^ goodness-of-fit. They are presented in the figures in the supplemental materials (Figs. S1–S7). A typical model for the competition between winter wheat (W) and blackgrass (B) occurred in two variants: model IIa, which represents the higher relative yield of B, and model IIb, when the higher relative yield was attributed to W (Fig. 4).

**Figure 5:**
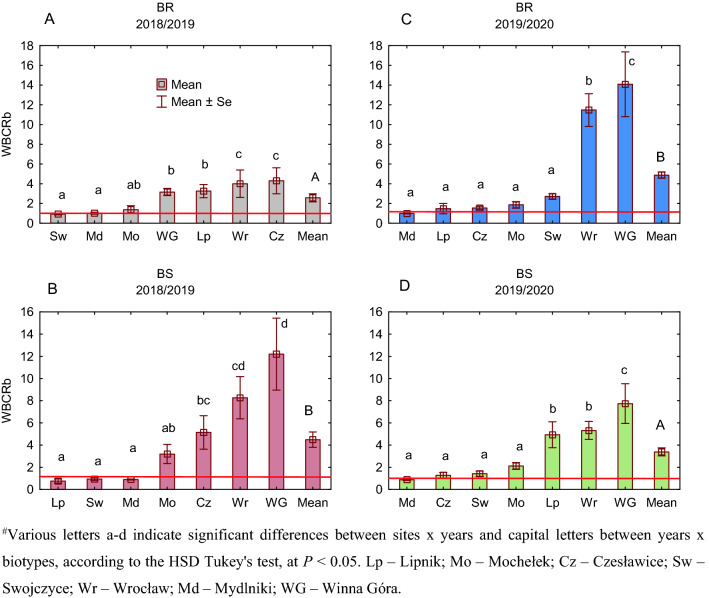
The competitive ratio for biomass production (WBCRb) between winter wheat and herbicide-resistant (BR)/susceptible (BS) blackgrass in the years of study for seven sites in Poland. Competitive ratio values < 1 indicate that blackgrass is more competitive than wheat.

**Figure 6:**
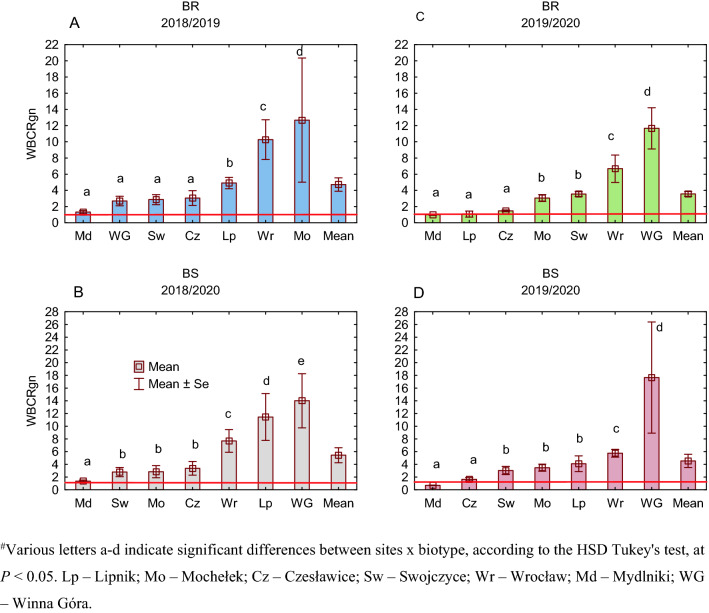
The competitive ratio for grain numbers (WBCRgn) between winter wheat and herbicide-resistant (BR)/susceptible (BS) blackgrass in the years of study for seven sites in Poland. Competitive ratio values < 1 indicate that blackgrass is more competitive than wheat.

The average competitive ratios for both biomass (WBCRb) and grain number (WBCRgn) were similar for the susceptible and resistant B biotypes in the competition with W (Figs. 5 and 6). However, in terms of WBCRb, there was a significant difference between seasons, with the R biotype being less competitive in 2018/2019 than in 2019/2020. Stronger competitiveness in biomass production was attributed to BR in the five locations in 2019/2020 (Mydlniki, Lipnik, Czesławice, Mochełek and Swojczyce) (Fig. 5C), while in 2018/2019, it was only at two locations, i.e. Swojczyce and Mydlniki (Fig. 5A). In 24 cases across Poland, W was more competitive than B in the mixture, as the RY (relative yield) of W was higher in the mixture (convex line) than the RY of B (concave line)—Model IIb (Fig. 4). Mydlniki was the only site where B, both R and S biotypes, was more competitive in the mixture with W, or no competitiveness occurred between both species in both seasons (Fig. S7) The competitive ratios for Mydlniki for the WBCRgn and WBCRb were low, 0.7–1.4 and 0.9–1.0, respectively (Figs. 5 and 6). Additionally, in the 2018/19 season, both the S and R biotypes in Swojczyce were more competitive toward W in the WBCRgn (Fig. 6A,C).

### Principle component analysis and cluster analysis

**Figure 7:**
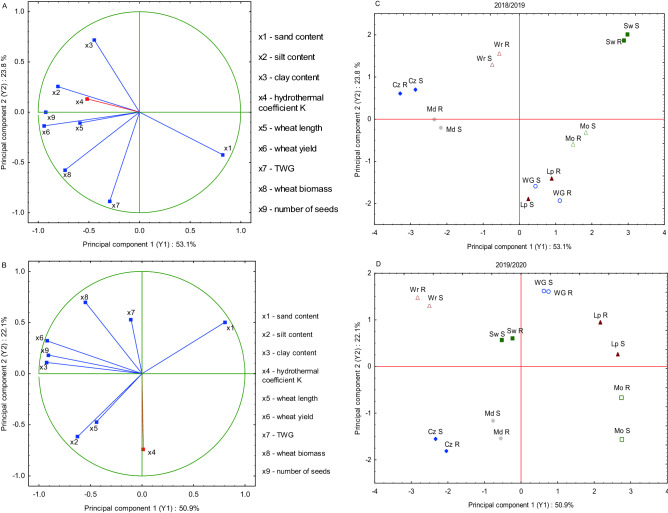
Projection of environmental characteristics and winter wheat characteristics growing in the mixtures with blackgrass resistant (R) and susceptible (S) biotypes in the seasons 2018/19 (**A**,**C**) and 2019/20 (**B**,**D**) on two principal components. Sites of experiments: *Lp* Lipnik, *Mo* Mochełek, *Md* Mydlniki, *Cz* Czesławice, *Sw* Swojczyce, *WG* Winna Góra, *Wr* Wrocław. *TGW* thousand grain weight, *K* hydrothermal coefficient.

The relationships between all traits of winter wheat W grown with BR and BS, as well as the environmental parameters, were calculated using principal multidimensional component (PCA) and cluster (CA) analyses separately for the 2018/19 (Fig. 7A,C) and 2019/20 (Fig. 7B,D) seasons, as the variation in years was in interaction with sites or biotype. Three characteristics of soil (sand, silt, and clay contents) were uniform in the two seasons, while the hydrothermal coefficients K and the W parameters varied in both seasons. In 2018/19, the first component (Y1) explained 53.1% of the total variance. It was significant for the silt content (× 2) and plant biomass (× 8), seed yield (× 6), and number of seeds (× 9) in the same direction, while the content of sand (× 1) was significant in the opposite direction: Y1 = 0.84x_1_ − 0.83x_2_ − 0.44x_3_ − 0.63x_4_ − 0.65x_5_ − 0.90x_6_ − 0.25x_7_ − 0.70x_8_ − 0.90x_9_. (× 1 − × 9 correspond to the characteristics in Fig. 7A). The second principal component (Y2) explained 23.8% of the total variance (Y_2_ = − 0.39x_1_ + 0.29x_2_ + 0.66x_3_ + 0.23x_4_ − 0.15x_5_ − 0.16x_6_ − 0.91x_7_ − 0.62x_8_ + 0.06x_9_), and it was significant for the thousand grain weight (TGW, × 7) (Fig. 7A). The projection of seven locations across Poland, where the BS/BR mixtures with W were planted, displayed two distinctive groups, i.e., Lipnik, Mochełek, Winna Góra, and Swojczyce, highly related to the sandy soil (mean = 76.63%) and low moisture (K = 1.1) in 2018/19 (Fig. 7C). Therefore, the alternation in significantly lower seed number (111.4) and seed yield (5.06) was found (Table 4). The second group consisted of sites, e.g., Czesławice, Wrocław, and Modlniki, where the W yield was significantly higher due to heavier soils (silt content = 49.25) and a higher hydrothermal coefficient (K = 1.97), so the seed number amounted to 245.1 and the yield amounted to 10.19 (Table 4).

**Table 4:** The means of winter wheat characteristics and environmental parameters in the two groups of experimental sites according to the *k*-means analysis.

x_i_	Characteristic	Year	Group 1	Group 2	*P*
			Lp S; Lp R; Mo S Mo R Sw S; Sw R; WG S; WG R	Cz S; Cz R; Wr S; Wr R; Md S; Md R	
1	Sand (× 1)		76.63	36.93	***
2	Silt (× 2)		20.15	49.20	**
3	Clay (× 3)		3.28	13.87	***
4	Coefficient K (× 4)	2019	1.10	1.97	*
		2020	2.23	2.53	ns
5	Length (× 5)	2019	59.55	73.95	ns
		2020	68.28	79.72	ns
6	Yield (× 6)	2019	5.06	10.19	**
		2020	6.62	11.68	**
7	TGW (× 7)	2019	42.24	41.16	ns
		2020	43.43	42.87	ns
8	Biomass (× 8)	2019	16.22	23.64	ns
		2020	27.87	28.81	ns
9	Seeds number (× 9)	2019	111.4	245.1	***
		2020	162.1	278.4	**

Sites of experiments: *Lp* Lipnik, *Mo* Mochełek, *Md* Mydlniki, *Cz* Czesławice, *Sw* Swojczyce, *WG* Winna Góra, *Wr* Wrocław. Mixture with (R) resistant, (S) susceptible biotype of blackgrass; *TGW* thousand-grain weight, *K* hydrothermal coefficient, *P* probability, *0.05, **0.01, ***0.001, *ns* not significant.

In the second season, the first principal component Y1 explained 50.9% of the total variance (Y_1_ = 0.92x_1_ − 0.62x_2_ − 0.80x_3_ + 0.02x_4_ − 0.44x − 0.92x_6_ − 0.11x_7_ − 0.55x_8_ − 0.91x_9_), and the second principal component Y2 explained 22.1% of the total variance of nine characteristics (Y_2_ = 0.55x_1_ − 0.91x_2_ + 0.16x_3_ − 0.75x_4_ − 0.51x_5_ + 0.31x_6_ + 0.67x_7_ + 0.71x_8_ + 0.18x_9_ (× 1 −  × 9 correspond to the characteristics in Fig. 7B). The projection of seven locations where W was planted in the mixtures with BS or BR configured a symmetric distribution on Y1 and Y2 (Fig. 7D). At sites Czesławice, Wrocław and Mydlniki, according to the heavy soil, the W yield parameters were significantly higher than those at other sites, e.g., Lipnik, Mochełek, Swojczyce and Winna Góra, despite the uniform hydrothermal conditions in 2019/20 (Table 4).

## Discussion

In Poland, blackgrass herbicide resistance has been recorded mainly in the northern and northeastern parts (Pomerania, Warmia-Mazury) and the Lower Silesia region^24,43^. The resistant biotype tested in our study had mutations at single positions within the ALS and ACCase sequences. In the ALS amino acid the changes at position P197 were identified. P197T was previously reported in *A. myosuroides* plants^44,45^, while P197A was detected in the *Lolium rigidum* and *Conyza canadensis* populations^46,47^. The W1999L mutation found in the ACCase sequence was also acknowledged to confer resistance to the ACCase-inhibiting herbicide fenoxaprop in *Alopecurus japonicus*^48^.

Various modeling approaches might be employed to examine the effectiveness of integrated weed management, reducing the need for more expensive and cumbersome long-term in situ experimentation^49^. We conducted short-term (two growing seasons) experiments in situ in the replacement series model as a tool to investigate the competitiveness between blackgrass-blackgrass and wheat-blackgrass with environmental factors and blackgrass herbicide resistance.

Within two years of study, the tested blackgrass biotypes, herbicide-susceptible (BS) and herbicide-resistant (BR), displayed different competitiveness with each other and with winter wheat. Based on two competitive ratios, i.e., plant biomass and the number of grains produced by the tested plants, we could draw main competitive relations, significant from biological and agronomic points of view^50^, in different sites across Poland. The sites in this experiment were diversified in terms of soil conditions, from the lightest loamy sands (Lipnik, Mochełek), sandy loams (Winna Góra, Swojczyce), medium-heavy sandy clay loam (Wrocław), to heavy and compacted silt loams (Mydlniki, Czesławice). Additionally, two vegetative seasons during the studies displayed contrasting weather conditions; the first season was dry in most of the sites, whereas the other season was humid.

In the BR-BS experiment, on average, the BS biotype was more competitive toward the BR biotype, which was expressed by the values of the competitive ratio greater than one. This is consistent with other authors' findings, who point out that herbicide-susceptible weed biotypes perform better than resistant ones in the absence of herbicide selective pressure. For example, under drought stress, the nontarget site-resistant biotype of blackgrass germinated less than the susceptible biotype^51^. In other studies, the blackgrass biotype with enhanced metabolism resistance to ALS inhibitors produced longer tillers than the susceptible plants during vegetative growth. However, there was a reproductive cost of resistance, i.e., 27% fewer seed heads per plant and a 23% reduction in total seed head length^52^. The worse performance of herbicide-resistant biotypes, the so-called resistance penalty, was also observed in other herbicide-resistant weed species^53–55^. Our study also showed site-specific correlations between both competitive ratios of BR and BS biotypes, especially in the humid season. We found that the content of sand affected the competitive relations between the BS-BR biotypes during the humid season. Specifically, the more sand there was in the soil, the higher the competitiveness of the BR biotype toward the BS biotype, and vice versa. In our opinion, this finding suggests the existence of internal plasticity of blackgrass biotypes that change their intraspecies competitive efforts depending on the site-specific conditions.

The diversified soil and weather conditions also affected the studied parameters of winter wheat (seed number and biomass), as was observed in the wheat (W) – barnyardgrass (BS/BR) experiment. The multivariate data mining at each site and BS/BR mixtures with winter wheat explained the division into two distinctive in situ groups. The first group was highly related to sandy soil and low moisture. In contrast, the second group had heavier soils of higher moisture where wheat productivity was significantly higher, as confirmed in the studies by^56^. In our study, wheat cv. Arkadia has been shown to suppress blackgrass at most study sites, particularly in the humid season of 2019/20 and regardless of the herbicide-susceptibility level of B. Increasing wheat competitiveness against weeds through a combination of breeding and planting design (planting density, row spacing, and orientation) has a strong potential to reduce weed-induced yield losses in wheat^30,57^. Sardana^58^ declared that cultivars possessing traits such as fast germination and growth, high biomass, and large leaf area have a competitive advantage over weeds. Lazzaro^59^ listed four traits linked to the competitive ability of wheat against weeds, i.e., aboveground biomass before stem elongation, tillering index, plant height, flag leaf morphology, and two production-related traits (grain yield and thousand-kernel weight). The combined effect on wheat-blackgrass interactions was also studied by Andrew and Storkey^60^ using the INTERCOM model parameterized for two wheat cultivars with contrasting competitive ability and simulations run across 10 years at different crop densities and two sowing dates. The authors found that sowing date, sowing density, and cultivar choice largely work in a complementary fashion, allowing enhanced competitive ability against weeds when used in combination. However, the relative benefit of choosing a more competitive cultivar decreases at later sowing dates and higher crop densities. Perhaps another important element in the competitiveness of W against B in our study that supported W competitiveness was the time of W emergence, which was in the vast majority of sites earlier than the emergence of B, or at least both species emerged on the same day. As other studies confirm, a delay in weed emergence to wheat emergence enhances wheat competitiveness toward weeds and a final wheat grain yield^42,61–63^.

The observed W-B competition pattern exception was noted only in two sites, namely, Mydlniki and Czesławice (south and south-eastern Poland), located on heavy, clay soils, where B was more competitive toward W during humid seasons. Blackgrass also takes advantage of environmental conditions, such as clay content and moisture in the soil^6,10^, which could support its competitiveness with W at both sites.

## Conclusions

Our study showed all the competitive replacement series models between blackgrass—blackgrass and winter wheat—blackgrass of different herbicide susceptibilities (R—resistant, S—susceptible). There were site-specific changes in the blackgrass R and S competitive abilities, depending on the sand soil content, supporting the thesis about blackgrass plasticity depending on environmental conditions. Winter wheat in our study was a strong competitor for blackgrass; only in three out of a total of 28 cases was blackgrass more competitive toward wheat. One of the winter wheat characteristics that could affect this relationship was that wheat emerged 15–0 days earlier than blackgrass. A more detailed understanding of belowground competition may also be required to increase the robustness of the predictions when water or nutrients are limiting.

## Materials and methods

### Plant material

Ethics approval and consent to participate. The collection of biotypes of blackgrass samples from plantations was permitted by local famers on survey. The plant collection and the study complied with local and national (Poland) regulations. The study was approved by the institutional research ethics committee of Institute of Plant Protection in Poznań, and written informed consent was obtained from each participant. All the methods in this manuscript were carried out in accordance with relevant guidelines and regulations.

Two biotypes of blackgrass (B) with contrasting susceptibility to herbicides were used in the two-year-long pot experiments at seven sites across Poland. Both biotypes were collected in Poland in 2017. The herbicide multiresistant (BR) biotype was from western Poland, voivodship Lubuskie, from winter wheat. The susceptible biotype (BS) was from eastern Poland from the ruderal site. An initial level of herbicide resistance of both BR and BS biotypes was confirmed in two sets of greenhouse dose–response tests carried out in 2017 and is presented in Table 5.

**Table 5:** Characteristics of herbicide multiresistant (BR) and herbicide-susceptible (S) biotypes of blackgrass used in the pot experiments.

Biotype	Herbicide (HRAC group)				
	Fenoxaprop-P (HRAC A)	Pinoxaden (HRAC A)	Piroxulam (HRAC B)	Iodosulfuron (HRAC B)	Pendimathalin (HRAC K1)
R	RRR (> 2650)	RRR (684)	RRR (> 288)	RRR (> 3200)	RRR (> 51000)
S	S (< 51.0)	S (< 25.5)	S (< 12.5)	S (< 5.0)	S (< 300)

The numbers in brackets relate to the effective dose (g ha^−1^) of the active ingredient causing a 50% reduction in plant biomass (ED_50_).

^1^*S* susceptible.

^2^*RRR* highly resistant.

### Molecular analysis of the herbicide-resistant blackgrass biotype

Leaves from plants of the multiresistant BR biotype in the leaf development stage (BBCH 13–14) were ground in a mortar using liquid nitrogen. Genomic DNA was isolated using a NucleoSpin Plant II Mini kit for DNA from plants (Mecherey-Nagel, Düren, Germany). Blackgrass ALS and ACCase gene sequences were amplified by means of PCR. For the analysis of the ALS sequence, equal amounts of DNA isolated from two plants were pooled and used in PCR, while for the analysis of the ACCase sequence, DNA was isolated from one plant. The 50 µl reaction mixture contained 5.0 µl of 10X PfuUltra II reaction buffer (Agilent Technologies, Santa Clara, CA, USA), 0.5 µl of dNTPs mix (25 mM each), 1.0 µl of 10 µM forward primer and reverse primers (Table 2), 100 ng of genomic DNA, and 1 U of PfuUltra II Fusion HS DNA polymerase (Agilent Technologies). PCR was carried out in a Mastercycler nexus (Eppendorf, Hamburg, Germany). The PCR program consisted of the following steps: an initial denaturation at 95 °C for 2 min, 35 cycles of amplification: 20 s at 95 °C, 20 s at the temperatures listed in Table, and 30 s or 45 s at 72 °C for the amplification of ALS and ACCase sequences, respectively. The final elongation step was set to 2 min at 72 °C. The reaction products were separated with 1% gel electrophoresis followed by purification from the gel with a Wizard SV Gel and PCR Clean-Up System (Promega, Madison, WI, USA). ALS and ACCase sequence fragments were ligated to the pJET1.2 plasmid using a CloneJET PCR Cloning Kit (Thermo Fisher Scientific, Waltham, MA, USA) and cloned into DH10B *Escherichia coli* competent cells (Table 6). Three plasmids were isolated from *E. coli* cells using the NucleoSpin Plasmid (Mecherey-Nagel). Three plasmids containing inserts synthesized in each PCR were digested with BglII to confirm the presence of the insert and sequenced by Genomed (Warsaw, Poland). Sequencing data were analyzed using the BioEdit Sequence Alignment Editor 7.5.5^64^.

**Table 6:** Primer sequences used in this study.

Gene	Primer sequence (5′–3′)	Amplicon length (bp)	Annealing temperature (°C)	References
*ALS*	F: TACCCAAACCTACTCTCCCG	1919	61.6	^44^
	R: TGATCAGGCACATTGCACC			
*ACCase*	F: AGGACACGCAGAGGAACCT	2879	60.6	Designed in this study
	R: GCAGCTGCCTCAGAAGCCAA			

#### Description of the replacement-series pot experiments

**Figure 8:**
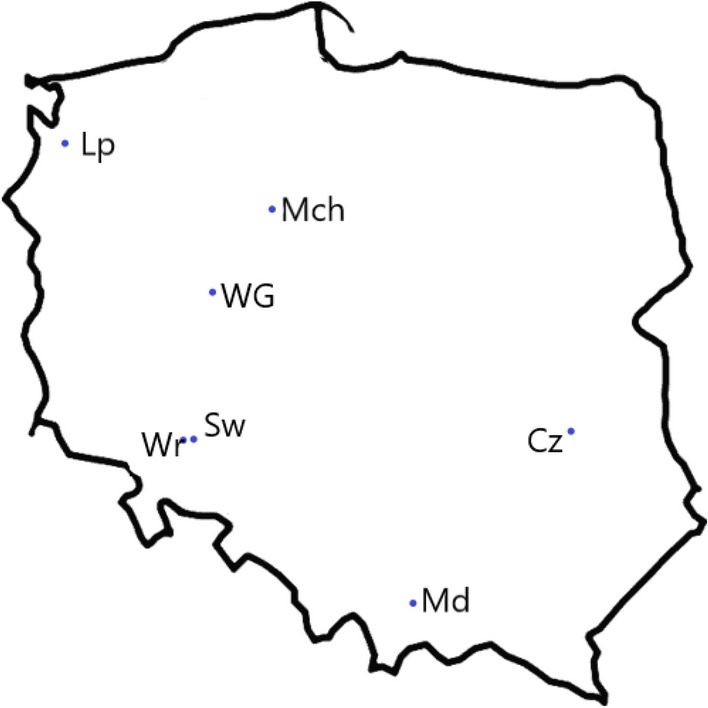
Study site distribution. *Lp* Lipnik; *Mo* Mochełek; *WG* Winna Góra; *Cz* Czesławice; *Sw* Swojczyce; *Wr* Wrocław; *Md* Mydlniki.

Following the replacement series competition model^65^, the pot experiments in field conditions were designed and set across Poland at six or seven sites (Fig. 8, Table 7). The experimental design was based on randomized blocks with three replications and took place in the next two growing seasons, 2018/19 and 2019/20.

**Table 7:** Coordinates and soil characteristics of the study sites.

Site	Latitude	Longitude	Particles (%)			Texture	N^1^	P	K	OM	pH
			Sand	Silt	Clay						
Lipnik	53°34′ N	14°95′ E	85.4	14.0	0.6	Loamy sand	0.11	232	301	2.2	6.4
Mochełek	53°20′ N	17°86′ E	84.7	14.3	1.1	Loamy sand	0.17	238	296	2.6	6.6
Winna Góra	52°12′ N	17°26′ E	70.4	26.3	3.4	Sandy loam	–	137	162	1.1	5.5
Czesławice	51°18′ N	22°16′ E	15.8	72.6	11.6	Silt loam	0.12	158	196	1.5	6.4
Swojczyce	51°06′ N	17°08′ E	66.0	26.0	8.0	Sandy loam	0.51	128	125	1.2	6.5
Wrocław	51°04′ N	17°02′ E	56.0	23.0	21.0	Sandy (clay) loam	0.70	182	197	1.2	6.2
Mydlniki	50°07′ N	19°84′ E	39.0	52.0	9.0	Silt loam	0.07	173	196	1.1	6.3

^1^*N* nitrogen (%), *P* phosphorus (mg kg^−1^), *K* potassium (mg kg^−1^), *OM* organic matter (%).

At each of the sites, pots (7 l vol., 0,0075 m^2^ area) were dug into the soil at a distance of 0.5 m, and ca. A total of 2.5 cm of pots remained above the soil surface. Subsequently, they were filled with local arable soil from the 0–30 cm layer. Prior to placing it in the pots, the soil was sieved through a metal mesh to remove stones and other larger impurities. Soil samples were taken for detailed texture and chemical analyses (Table 7). Black foil was placed in the area between pots, and the whole experiment was protected from insects and birds with a fine plastic net.

The layout of pots and the distribution of plants in a pot remained the same at every site. The competitive effects of BR and BS biotypes were studied in two proportions of plants R4:S6 and R6:S4 at six sites, i.e., Lipnik, Mochełek, Czesławice, Swojczyce, Wrocław, and Mydlniki. The competitive effects of BR or BS were tested against winter wheat (W) cv. Arkadia (breeder: HR Danko PL) at all seven sites. The proportions of winter wheat W and BR were in the ratios of W10:R0, W8:R2, W6:R4, W4:R6, W2:R8, and W0:R10. The same ratios were used for the W and BS plants: W10:S0, W8:S2, W6:S4, W4:S6, W2:S8, and W0:S10.

Seeds of the W, BR, and BS biotypes were sown on the same day, optimal for W (Table 8). The grains of B were sown at a depth of 0.5 cm, and W was sown at a depth of 2 cm, with a few additional grains per spot. The sowing spot of each plant was marked.

**Table 8:** Dates of winter wheat sowing and harvest.

Study site	2018/19		2019/20	
	Sowing	Harvest	Sowing	Harvest
Lipnik	18.10.2018	10.07.2019	17.10.2019	19.07.2020
Mochełek	17.10.2018	03.07.2019	17.10.2019	08.07.2020
Winna Góra	18.10.2018	11.07.2019	15.10.2019	23.07.2020
Czesławice	09.10.2018	24.07.2019	08.10.2019	23.07.2020
Swojczyce	13.10.2018	17.07.2019	17.10.2019	28.07.2020
Wrocław	25.10.2018	18.07.2019	08.10.2019	09.07.2020
Mydlniki	04.10.2018	13.06.2019	06.10.2019	21.07.2020

In the spring, the density of W and B plants was regulated according to the layout. During that time, N fertilization was applied in 30.0 g NH_4_NO_3_ m^−2^; 50% of the dose was applied at the beginning of vegetation, and the remaining dose was applied at wheat shooting (BBCH 31–33). Wheat and blackgrass were harvested at full-grain maturity (BBCH 97–99). At harvest, a fresh mass of plants, both W and B, was weighed. Wheat plants were measured with a ruler from the base of shoots up to the highest ear's tip. All grains of W/B plants were counted from the pot, and their fresh mass was weighed and calculated per plant for statistical analysis. Based on three samples of 100 grains, the 1000 grain weight (TGW) of W and B was calculated.

### Weather conditions

Weather data, i.e., precipitation and air temperature, were collected during the study from the nearest weather stations (Tables S1 and S2). The weather data for the months from winter wheat sowing till harvesting, with temperatures > 0 °C, namely, October–November 2018 and April–July 2019, October–November 2019, and April–July 2020, were valorized using the hydrothermal coefficient (*K*) according to the equation:

1 $$K = 10{\text{P}}/{\text{t}},$$

where *P* is the precipitation total and *t* is the sum of the daily mean air temperature values. The classification for Poland's temperate climate is *K* < 1.3 moderately dry seasons; 1.3–1.6 optimum; and > 1.6 humid season^66^. The calculated *K* values are presented in Table 9.

**Table 9:** Hydrothermal coefficient (*K*) calculated for the seasons 2018/19 and 2019/20 for the study sites.

Study site	2018/19	Classification	2019/20	Classification
Lipnik	1.2	Relatively dry	2.7	Humid
Mochełek	0.7	Dry	3.0	Humid
Winna Góra	1.0	Dry	0.8	Dry
Czesławice	1.5	Optimal	4.0	Humid
Swojczyce	1.5	Optimal	2.4	Humid
Wrocław	1.1	Relatively dry	1.6	Optimal
Mydlniki	3.3	Humid	2.0	Humid

### Statistical methods

Two parameters, namely, fresh plant biomass and the number of seeds per plant, were measured for both winter wheat W and blackgrass B. The blackgrass competitive ratio (BCR) was calculated for the mixtures of susceptible blackgrass (BS) with resistant blackgrass (BR), representing the comparative growth based on plant fresh biomass (BCRb) and the number of grains (BCRgn). The competitive ratio between wheat and blackgrass WBCR was calculated separately for the mixtures of W and BS and W and BR.

The BCR and WBCR were calculated according to the formula described by Hoffman and Buller^67^:

2 $${\text{BCR}} = \left( {\left( {1\;{\text{p}}} \right)/{\text{p}}} \right)/\left( {{\text{RY}}_{{{\text{BS}}}} /{\text{RY}}_{{{\text{BR}}}} } \right),$$

where RY is the relative yield and p is the proportion of biotype.

If the competitive ratio BCR < 1, the R biotype is more competitive toward the S biotype; if BCR > 1, the S biotype is more competitive toward the R biotype.

3 $${\text{WBCR}} = \left( {\left( {{1}\;{\text{p}}} \right)/{\text{p}}} \right)/\left( {{\text{RY}}_{{\text{W}}} /{\text{RY}}_{{\text{B}}} } \right),$$

where RY is the relative yield and p is the proportion of species.

If the competitive ratio WBCR < 1, B is more competitive toward W; if WBCR > 1, W is more competitive toward B.

The RY and the total relative yield (TRY) for all the measured parameters of B and W were calculated according to the formulas:

4 $${\text{RY}}_{{\text{W}}} = \left( {\text{p}} \right)\;\left( {{\text{W}}_{{{\text{mix}}}} /{\text{W}}_{{{\text{mon}}}} } \right),$$

5 $${\text{RY}}_{{\text{B}}} = \left( {{\text{p}} - {1}} \right)\;\left( {{\text{B}}_{{{\text{mix}}}} /{\text{B}}_{{{\text{mon}}}} } \right),$$

6 $${\text{TRY}} = {\text{RY}}_{{\text{W}}} + {\text{RY}}_{{\text{B}}} ,$$

The analysis was performed for the replacement series experiment^63^. where RY_W_ is the relative yield of W; RY_B_ is the relative yield of B; p is the proportion of species; W_mix_ is the value of the W parameter analyzed for the mixture; W_mon_ is the value of the W parameter analyzed for the monoculture; B_mix_ is the value of the B parameter analyzed for the mixture; B_mon_ is the value of the B parameter analyzed for the monoculture; and TRY is the total relative yield. The RY values were measured for both W and B and were averaged for a single plant.

The biomass and grain yield or seed number, calculated into RY and TRY, were presented as graphs and fitted into one of the five competition models according to Radosevich^68^. The most explanatory models were determined by using the χ^2^ goodness-of-fit^69^, giving the best fit for all tested sites and biotypes. If RY is a straight line, it denotes no competition; a convex line shows a benefit to species; a concave line represents a loss to species. If TRY equals 1 (straight line), there is competition for the same resources between both species/biotypes. If TRY is greater than 1 (convex), there is no competition since the demand does not exceed the resources. If TRY is less than 1 (concave), an antagonism resulting in a mutual loss to the species involved becomes apparent^67^.

ANOVA of two models was performed, i.e. the two-way model for the year × site of plants to calculate the BCRgn and BCRb in R/S blackgrass and the three-way model for the year × site × biotype to calculate the WBCRgn and WBCRb in wheat/blackgrass. Data from the indexes were transformed to a normal distribution. The post hoc HSD Tukey test was used for the means separation. The r-Pearson correlation coefficient was calculated for relations between BCRgn/WBCRgn and BCRb/WBCRb, the relations between the hydrothermal K coefficient and BCRgn/WBCRgn and BCRb/WBCRb and the relations between sand [%] and BCRgn/WBCRgn and BCRb/WBCRb. The exploration technique of principal component analysis (PCA) was used to explain the multidimensional diversity of W and environmental variables in terms of the first two components. Five biometrical parameters of W, i.e. mean length and fresh biomass of the plant, number of seeds per plant, yield of seeds per plant, and TGW (1000 grain weight), and four environmental parameters, i.e. The hydrothermal coefficient K and soil texture were analyzed using PCA and CA (*k*-means procedure).

The calculations were performed in the STATISTICA 13.0 program (TIBCO Software Inc.).

## Supplementary Information

Supplementary Figures.

Supplementary Tables.

## Data Availability

Data available from Zenodo repository under https://zenodo.org/record/5910336.
