# Supplementary material for: Intra- and interspecies competition of blackgrass and wheat in the context of herbicidal resistance and environmental conditions in Poland
Source: Sci Rep. 2022 May 24;12:8720. doi: 10.1038/s41598-022-12777-2 (PMC9130282; doi:10.1038/s41598-022-12777-2)
Supplement: Supplementary file 1 — Supplementary Figures. [file 41598_2022_12777_MOESM1_ESM.docx]

**Supplementary material**

| 2018/2019 – S | | |
| --- | --- | --- |
| Model IIb | Model IV | |
|  |  | |
| 2018/2019 – R | | |
| Model IIb | Model IIb | |
|  |  |  |
| 2019/2020 – S | | |
| Model IIb | Model IIb | |
|  |  | |
| 2019/2020 – R | | |
| Model IIb | Model IIb | |
|  |  |  |

Figure S1. The substitutive competition model for the relative grain number (RYgn) and biomass yield (RYb) of winter wheat (W) and blackgrass (B) in the 2018/2019 and 2019/2020 seasons in Lipnik. R- blackgrass with a multiple herbicide resistance, S- susceptible blackgrass. Legend: □ black solid line – W; ♦ black dotted line – B, ● grey dotted line – W + B

| 2018/2019 – S | | |
| --- | --- | --- |
| Model IIb | Model III | |
|  |  | |
| 2018/2019 – R | | |
| Model IIb | Model III | |
|  |  |  |
| 2019/2020 – S | | |
| Model IV | Model IIb | |
|  |  | |
| 2019/2020 – R | | |
| Model IV | Model IIb | |
|  |  |  |

Figure S2. The substitutive competition model for the relative grain number (RYgn) and biomass yield (RYb) of winter wheat (W) and blackgrass (B) in the 2018/2019 and 2019/2020 seasons in Mochełek. R- blackgrass with a multiple herbicide resistance, S- susceptible blackgrass. Legend: □ black solid line – W; ♦ black dotted line – B, ● grey dotted line – W + B

| 2018/2019 – S | | |
| --- | --- | --- |
| Model IIb | Model III | |
|  |  | |
| 2018/2019 – R | | |
| Model I | Model III | |
|  |  |  |
| 2019/2020 – S | | |
| Model IIb | Model IIb | |
|  |  | |
| 2019/2020 – R | | |
| Model IIb | Model IIb | |
|  |  |  |

Figure S3. The substitutive competition model for the relative grain number (RYgn) and biomass yield (RYb) of winter wheat (W) and blackgrass (B) in the 2018/2019 and 2019/2020 seasons in Winna Góra. R- blackgrass with a multiple herbicide resistance, S- susceptible blackgrass. Legend: □ black solid line – W; ♦ black dotted line – B, ● grey dotted line – W + B

| 2018/2019 – S | | |
| --- | --- | --- |
| Model IIb | Model IIb | |
|  |  | |
| 2018/2019 – R | | |
| Model IIb | Model IIb | |
|  |  |  |
| 2019/2020 – S | | |
| Model IIb | Model IV | |
|  |  | |
| 2019/2020 – R | | |
| Model I | Model IIb | |
|  |  |  |

Figure S4. The substitutive competition model for the relative grain number (RYgn) and biomass yield (RYb) of winter wheat (W) and blackgrass (B) in the 2018/2019 and 2019/2020 seasons in Czesławice. R- blackgrass with a multiple herbicide resistance, S- susceptible blackgrass. Legend: □ black solid line – W; ♦ black dotted line – B, ● grey dotted line – W + B

| 2018/2019 – S | | |
| --- | --- | --- |
| Model IV | Model IV | |
|  |  | |
| 2018/2019 – R | | |
| Model IV | Model IIb | |
|  |  |  |
| 2019/2020 – S | | |
| Model IIb | Model IIb | |
|  |  | |
| 2019/2020 – R | | |
| Model IIb | Model IIb | |
|  |  |  |

Figure S5. The substitutive competition model for the relative grain number (RYgn) and biomass yield (RYb) of winter wheat (W) and blackgrass (B) in the 2018/2019 and 2019/2020 seasons in Wrocław. R- blackgrass with a multiple herbicide resistance, S- susceptible blackgrass. Legend: □ black solid line – W; ♦ black dotted line – B, ● grey dotted line – W + B

| 2018/2019 – S | | |
| --- | --- | --- |
| Model IIb | Model III | |
|  |  | |
| 2018/2019 – R | | |
| Model IIb | Model III | |
|  |  |  |
| 2019/2020 – S | | |
| Model IIb | Model IV | |
|  |  | |
| 2019/2020 – R | | |
| Model IIb | Model IIb | |
|  |  |  |

Figure S6. The substitutive competition model for the relative grain number (RYgn) and biomass yield (RYb) of winter wheat (W) and blackgrass (B) in the 2018/2019 and 2019/2020 seasons in Swojczyce. R- blackgrass with a multiple herbicide resistance, S- susceptible blackgrass. Legend: □ black solid line – W; ♦ black dotted line – B, ● grey dotted line – W + B

| 2018/2019 – S | |
| --- | --- |
| Model IIb | Model III |
|  |  |
| 2018/2019 – R | |
| Model IIb | Model III |
|  |  |
| 2019/2020 – S | |
| Model IIa | Model IIa |
|  |  |
| 2019/2020 – R | |
| Model IIa | Model IIa |
|  |  |

Figure S7. The substitutive competition model for the relative grain number (RYgn) and biomass yield (RYb) of winter wheat (W) and blackgrass (B) in the 2018/2019 and 2019/2020 seasons in Mydlniki. R- blackgrass with a multiple herbicide resistance, S- susceptible blackgrass. Legend: □ black solid line – W; ♦ black dotted line – B, ● grey dotted line – W + B
