# Supplementary material for: Intra- and interspecies competition of blackgrass and wheat in the context of herbicidal resistance and environmental conditions in Poland
Source: Sci Rep. 2022 May 24;12:8720. doi: 10.1038/s41598-022-12777-2 (PMC9130282; doi:10.1038/s41598-022-12777-2)
Supplement: Supplementary file 2 — Supplementary Tables. [file 41598_2022_12777_MOESM2_ESM.docx]

Table S1. Sum of precipitation and mean temperatures during season 2018/19 in the sites of study.

| Month | Precipitation | | | | | | |  | Temperature | | | | | | |
| --- | --- | --- | --- | --- | --- | --- | --- | --- | --- | --- | --- | --- | --- | --- | --- |
|  | Lipnik | Mochełek | Winna Góra | Czesławice | Swojczyce | Wrocław | Mydlniki |  | Lipnik | Mochełek | Winna Góra | Czesławice | Swojczyce | Wrocław | Mydlniki |
| X | 16,2 | 27,4 | 21,1 | 41,3 | 46,1 | 32,2 | 52,6 |  | 13,4 | 10,5 | 11,3 | 9,2 | 10,5 | 10,6 | 8,2 |
| XI | 5,9 | 9,8 | 10,9 | 15,2 | 12,5 | 10,1 | 42,5 |  | 8,4 | 5,1 | 5,5 | 3,9 | 5,3 | 5,3 | 3,3 |
| XII | 70,0 | 46,2 | 40,6 | 70,8 | 41,7 | 40,3 | 45,6 |  | 3,7 | 2,5 | 2,8 | -0,2 | 2,6 | 2,6 | -0,9 |
| I | 28,0 | 31 | 25,6 | 31,5 | 49,6 | 33,2 | 44,6 |  | -2,5 | 0,2 | 0,1 | -3,4 | -0,2 | -0,2 | -0,9 |
| II | 32,0 | 17,6 | 12,6 | 14,6 | 27,4 | 25,0 | 13,1 |  | -1,6 | 4,0 | 3,5 | 2,5 | 3,2 | 3,5 | -2,7 |
| III | 37,5 | 0,0 | 32,9 | 27,1 | 23,9 | 27,7 | 21,2 |  | 3,4 | 2,7 | 6,6 | 5,5 | 6,5 | 6,7 | 1,4 |
| IV | 2,5 | 1,8 | 6,1 | 39 | 40,7 | 29,4 | 77,1 |  | 8,6 | 9,6 | 10,7 | 10,3 | 10,2 | 10,6 | 15,7 |
| V | 39,5 | 61,8 | 83,4 | 87 | 92,8 | 57,0 | 207,6 |  | 13,4 | 12,4 | 12,4 | 14,4 | 12,2 | 12,0 | 16,9 |
| VI | 35,0 | 11,2 | 2,1 | 11,2 | 23,3 | 8,8 | 22,8 |  | 16,8 | 19,4 | 22,8 | 22,9 | 22,5 | 22,3 | 18,8 |
| VII | 22,0 | 28,1 | 4,7 | 46,3 | 48,1 | 33,0 | 54,1 |  | 18,4 | 18,8 | 19,53 | 20 | 19,9 | 19,3 | 20,1 |

Table S2. Sum of precipitation and mean temperatures during season 2019/20 in the sites of study.

| Month | Precipitation | | | | | | |  | Temperature | | | | | | |
| --- | --- | --- | --- | --- | --- | --- | --- | --- | --- | --- | --- | --- | --- | --- | --- |
|  | Lipnik | Mochełek | Winna Góra | Czesławice | Swojczyce | Wrocław | Mydlniki |  | Lipnik | Mochełek | Winna Góra | Czesławice | Swojczyce | Wrocław | Mydlniki |
| X | 53,0 | 42,2 | 1,0 | 37 | 32,1 | b.d | 37,7 |  | 9,6 | 10,9 | 11,2 | 12,6 | 10,4 | b.d | 8,7 |
| XI | 47,0 | 59,9 | 0,8 | 56,3 | 31,6 | 1,9 | 42,4 |  | 4,0 | 6,1 | 6,68 | 6,6 | 6,9 | 6,3 | 3,7 |
| XII | 34,0 | 30,2 | 0,8 | 46,3 | 19,6 | 12,9 | 38,0 |  | -0,1 | 3,4 | 3,79 | 2,6 | 3,4 | 3,5 | -0,8 |
| I | 35,5 | 41,9 | 11,1 | 14,1 | 14,8 | 16,3 | 15,2 |  | 3,7 | 3,3 | 3,14 | 1,2 | 2,0 | 2,4 | -2,1 |
| II | 62,0 | 47,8 | 52,4 | 76,5 | 66,7 | 66,2 | 41,3 |  | 4,7 | 4,7 | 4,93 | 3,2 | 5,3 | 5,1 | -0,5 |
| III | 44,0 | 35,3 | 15,7 | 26 | 23,4 | 17,7 | 15 |  | 4,4 | 5,1 | 5,27 | 4,7 | 5,1 | 5,3 | 9,3 |
| IV | 13,0 | 1,5 | 1,4 | 19 | 9,6 | 5,7 | 8,1 |  | 8,8 | 9,7 | 9,59 | 8,6 | 9,3 | 9,7 | 10,8 |
| V | 50,5 | 43,9 | 2,5 | 111,4 | 74,6 | 43,7 | 91,2 |  | 11,2 | 12,2 | 11,8 | 11,2 | 11,9 | 11,6 | 12 |
| VI | 16,0 | 210,1 | 9,6 | 170,2 | 191,5 | 53,1 | 87,4 |  | 17,9 | 18,8 | 18,1 | 17,9 | 17,8 | 17,8 | 21 |
| VII | 25,5 | 81,3 | 14,0 | 67,8 | 19,4 | 5,4 | 72,9 |  | 17,8 | 19,3 | 18,64 | 18,6 | 19,0 | 18,6 | 19,6 |
